# Supplementary material for: Mechanisms governing the pioneering and redistribution capabilities of the non-classical pioneer PU.1
Source: Nat Commun. 2020 Jan 21;11:402. doi: 10.1038/s41467-019-13960-2 (PMC6972792; doi:10.1038/s41467-019-13960-2)
Supplement: Supplementary file 7 — Source data [file 41467_2019_13960_MOESM7_ESM.zip › Source_Data/FigureS2/FigureS2B_MotifScanOutput/KmeansCluster1/homerResults/motif5.similar.html]

motif5

## Information for motif5

C
T
A
G
C
G
T
A
C
T
G
A
A
C
T
G
C
T
G
A
T
C
A
G
A
C
T
G
C
G
T
A
  
Reverse Opposite:  

A
C
G
T
A
G
T
C
A
G
T
C
G
A
C
T
A
G
T
C
A
G
C
T
A
C
G
T
A
G
T
C
  

|  |  |
| --- | --- |
| p-value: | 1e-392 |
| log p-value: | -9.029e+02 |
| Information Content per bp: | 1.857 |
| Number of Target Sequences with motif | 2039.0 |
| Percentage of Target Sequences with motif | 40.05% |
| Number of Background Sequences with motif | 5502.6 |
| Percentage of Background Sequences with motif | 13.87% |
| Average Position of motif in Targets | 101.4 +/- 48.5bp |
| Average Position of motif in Background | 102.8 +/- 91.0bp |
| Strand Bias (log2 ratio + to - strand density) | -0.1 |
| Multiplicity (# of sites on avg that occur together) | 2.04 |
| Motif File: | file (matrix) reverse opposite |

### Similar de novo motifs found

|  |  |  |  |  |  |  |  |
| --- | --- | --- | --- | --- | --- | --- | --- |
| Rank | Match Score | Redundant Motif | P-value | log P-value | % of Targets | % of Background | Motif file |
| 1 | 0.762 | A T C G C G T A C T A G T C A G T C G A C G T A A C T G C G T A C T A G T C A G G T C A C T G A T C A G | 1e-379 | -873.497527 | 22.57% | 4.27% | motif file (matrix) |
| 2 | 0.858 | A C G T A G T C A G C T A C G T A G T C A C G T A C G T | 1e-269 | -621.461093 | 46.47% | 22.46% | motif file (matrix) |
| 3 | 0.838 | A G C T A C G T A T G C A G C T A C G T A G T C A C G T A C G T A G T C | 1e-124 | -286.212093 | 14.52% | 4.93% | motif file (matrix) |
| 4 | 0.762 | T C A G C T G A C T A G C T A G G T C A C T G A T C A G C G T A A C T G T C A G C G T A A C T G G C T A | 1e-99 | -229.872235 | 6.32% | 1.19% | motif file (matrix) |
| 5 | 0.802 | C T G A C T A G C T A G C T G A T C A G T C A G C T G A C T A G C T A G C T G A | 1e-65 | -149.838715 | 4.30% | 0.85% | motif file (matrix) |
